# Regenerative Approaches to Enhance the Skin Microenvironment and Boost Aesthetic Efficacy: A Narrative Review

**DOI:** 10.3390/ijms27114716

**Published:** 2026-05-23

**Authors:** Valéria Dal Col, Fábio Fernandes Ribas, Rodrigo Pinheiro Araldi

**Affiliations:** 1 Protocoll Clinique, Vitoria 29055-260, ES, Brazil; fabioribas@gmail.com; 2 Graduate Program in Endocrinology and Metabology, Paulista School of Medicine (EPM), Federal University of São Paulo (UNIFESP), São Paulo 04203-062, SP, Brazil; 3 BioDecision Analytics, São Paulo 01451-917, SP, Brazil

**Keywords:** regenerative aesthetic, skin bed preparation, cellular senescence, NAD^+^ boosters, mitochondrial bioenergetics

## Abstract

Aesthetic medicine is shifting from symptomatic correction to biological structural restoration. Regenerative aesthetics represents a frontier in dermatology, focusing on the restoration of the skin microenvironment to enhance cellular vitality and tissue resilience. Central to this approach is the concept of “skin bed preparation”, a strategic priming phase designed to optimize the physiological terrain before the delivery of advanced aesthetic interventions. This review explores the molecular and cellular mechanisms by which skin bed preparation modulates the extracellular matrix (ECM) and the dermal niche to maximize the efficacy of subsequent treatments and promote long-term skin longevity. Evidence suggests that biostimulatory priming utilizing senolytics, senomorphics, mitochondrial, and/or epigenetic rejuvenators rehabilitates the fibroblast–collagen interactome. By reducing oxidative stress and chronic low-grade inflammation, these preparatory steps transition the skin from a catabolic to an anabolic state. This metabolic reset ensures that subsequent procedures, such as laser therapy, injectable fillers, encounter a responsive cellular environment, resulting in superior collagen induction and prolonged clinical outcomes. Optimizing the skin microenvironment via regenerative aesthetics is not merely an adjunctive step but a fundamental requirement for therapeutic success. Integrating skin bed preparation into clinical protocols provides a synergistic framework that enhances immediate procedural results while addressing the underlying hallmarks of skin aging, ultimately redefining the trajectory of skin health and longevity.

## 1. The Biology Behind Better Aesthetic Results

The global medical aesthetics market is experiencing significant structural expansion, with projections indicating a valuation of approximately USD 19.05 billion by the end of 2026, sustained by a compound annual growth rate (CAGR) of 9% [1]. This growth is primarily driven by the democratization of non-invasive or minimally invasive procedures, which now account for most clinical interventions due to reduced downtime and high safety profiles [1]. Within this landscape, Brazil remains a pivotal epicenter, ranking as the world’s second-largest market for aesthetic procedures and a primary consumer of advanced dermo-cosmetic technologies [2].

The global increase in the aesthetic market has been driven not only by technological advances and expanding consumer access but also by the powerful influence of digital culture on perceptions of aging and beauty. Social media, high-resolution cameras, and algorithm-shaped beauty ideals have heightened sensitivity to facial changes and skin quality, creating a constant comparison between natural appearance and digitally enhanced images [3,4,5,6,7]. In Brazil, where aesthetic awareness is particularly strong, this digital exposure has intensified the demand for subtle, preventive, and harmonizing procedures that maintain youthfulness without altering personal identity [6]. As a result, aesthetic interventions increasingly function as tools for sustaining confidence and social presence in an image-saturated environment, reinforcing the broader shift toward minimally invasive and psychologically oriented aesthetic care. Thus, the contemporary pursuit of aesthetic procedures has transitioned from a niche luxury to a fundamental component of psychosocial health, with significant implications for an individual’s quality of life (QoL) and self-perception [2,8,9,10].

Evidence from 2024 and 2025 longitudinal studies suggests that aesthetic interventions, ranging from non-surgical dermal modulation (using laser therapy, injectable fillers, biostimulators, microneedling, and chemical peels) to surgical restructuring, yield quantifiable improvements in emotional stability, body image satisfaction, and social functioning [11]. This psychological benefit is particularly pronounced in procedures that align an individual’s external appearance with their internal body image, thereby alleviating sub-clinical levels of anxiety and depression [2,8,9].

However, the success of these outcomes is fundamentally dependent on a precise skin assessment to identify the most adequate treatment according to biological age and the skin’s intrinsic physiological capability to respond to the proposed protocol [12,13,14].

In this regard, the contemporary landscape of aesthetics is increasingly defined by the physiological optimization of the cutaneous environment prior to any interventional procedure [2,15,16]. Therefore, skin assessment and preparation are no longer considered auxiliary but fundamental, as the cellular signalling capacity and pre-treatment protocol directly dictate the healing velocity and the final aesthetic outcome [17,18,19,20].

Thus, conventional aesthetic, which traditionally focuses on correcting the visible consequences of aging by restoring volume, stimulating collagen, and producing rapid, appearance-centered results, has been replaced to regenerative longevity aesthetics. This emerging paradigm shifts the emphasis from superficial correction to the preservation of tissue function, the maintenance of intrinsic regenerative capacity, and the modulation of the biological mechanisms that drive cutaneous aging. By targeting cellular metabolism, mitochondrial efficiency, extracellular matrix integrity, and overall dermal homeostasis, regenerative longevity aesthetics seek to sustain long-term skin health and deliver outcomes that are not only aesthetically favorable but biologically durable.

Building on these considerations, the present narrative review aims to summarize current evidence on the critical role of skin preparation in maximizing the efficacy and aesthetic outcomes of clinical protocols. In the evolving field of regenerative aesthetics, terms such as ‘biopriming,’ ‘preconditioning,’ and ‘regenerative longevity aesthetics’ have frequently been employed as synonyms to describe the physiological optimization of the cutaneous environment. However, to establish a rigorous clinical nomenclature and avoid semantic ambiguity, this review adopts the consolidated term skin bed preparation.

To achieve this, the review examines the fundamental pathophysiological mechanisms underlying natural skin aging and delineates how targeted therapeutic interventions act upon each of these processes. For this, we focus on molecular decline, mitochondrial dysfunction, bioenergetic impairment, and the progression of cellular senescence, as well as emerging strategies that promote cellular longevity. By integrating these biological insights with practical approaches to pre-treatment optimization, this review seeks to support the development of more robust, efficient, and scientifically grounded aesthetic protocols capable of enhancing both procedural performance and long-term patient outcomes.

To support this narrative review, a comprehensive literature search was conducted across the PubMed, Scopus, and Google Scholar databases, covering publications primarily from the last decade. The search strategy employed keywords related to regenerative aesthetics, skin bed preparation, cellular senescence, mitochondrial bioenergetics, and multi-Omics technologies. This review integrates evidence from both preclinical (in vitro 2D and 3D cultures, skin-on-a-chip, and in vivo animal models) and clinical studies (longitudinal trials and biophysical skin assessments) to provide a translational perspective on skin aging and rejuvenation. In vitro studies were specifically utilized to elucidate molecular pathways, such as fibroblast activation and collagen synthesis, while in vivo and clinical data were prioritized to validate the efficacy and safety of regenerative interventions and skin bed preparation protocols.

## 2. Pathophysiology of Skin Aging: An Integrated Mechanistic Framework

Skin aging is a complex, multifactorial biological process driven by the interplay of intrinsic and extrinsic mechanisms that progressively compromise the structural, biochemical, and functional integrity of the cutaneous tissue [21,22,23,24].

Intrinsic aging, also known as chronological aging, is regulated by genetically programmed cellular events that unfold gradually over time (molecular decay) [23]. These include a decline in epidermal turnover (which turnover rate slows from 30–50% between our thirties and eighties) [25,26,27], reduced fibroblast proliferation [28,29], diminished synthesis of ECM components [30,31,32], and impaired barrier homeostasis [33,34,35].

Extrinsic aging, in contrast, results from cumulative environmental exposures such as ultraviolet (UV) radiation, pollution, tobacco smoke, and nutritional factors [36,37,38]. These external stressors amplify oxidative damage, inflammation, and matrix degradation, accelerating the visible and functional deterioration of the skin [36,37,38]. Together, intrinsic and extrinsic pathways lead to hallmark features of aged skin, including dermal thinning, collagen fragmentation, elastin fiber disorganization, increased transepidermal water loss, and dysregulated pigmentation [38].

Molecular decay represents a central hallmark of cutaneous aging, reflecting the progressive accumulation of structural and functional damage to DNA, proteins, lipids, and mitochondrial components [39,40]. This deterioration arises from the combined influence of intrinsic metabolic processes and extrinsic environmental exposures, both of which generate persistent oxidative and electrophilic stress [21,22]. Endogenous sources such as mitochondrial ROS, spontaneous hydrolytic reactions, and glycation progressively erode the integrity of biomolecules, while extrinsic factors, including UV radiation, pollution, and xenobiotics, amplify this burden. As these insults accumulate, the skin’s capacity to maintain proteostasis, genomic stability, and barrier function becomes increasingly compromised, establishing a biochemical environment that accelerates tissue decline [39,40].

A major driver of molecular decay is genomic instability, which emerges from both intrinsic replication-associated stress and extrinsic DNA-damaging agents [41]. During normal cell division, keratinocytes and fibroblasts experience replication-fork stress, characterized by stalled or collapsed replication forks due to nucleotide imbalance, oxidative lesions, or tightly bound protein–DNA complexes [42,43]. These events generate single- and double-strand breaks that, when inefficiently repaired, propagate mutations and chromosomal aberrations [41,44]. Extrinsic factors, particularly UV radiation, further intensify genomic damage through the formation of cyclobutene pyrimidine dimers (CPDs), 6-4 photoproducts, and oxidative base modifications such as 8-oxo-dG [45,46,47,48]. These lesions distort DNA structure, impede transcription and replication, and activate chronic DNA damage responses (DDR) [46,48]. Over time, persistent DDR signaling contributes to cellular senescence, impaired regenerative capacity, and the secretion of pro-inflammatory mediators that degrade the extracellular matrix [49,50,51]. Together, replication-fork stress and UV-induced lesions create a cumulative genotoxic load that undermines the skin’s ability to maintain genomic fidelity.

In parallel with direct molecular damage, age-related alterations in DNA methylation patterns, histone modifications, and chromatin accessibility disrupt the transcriptional programs required for epidermal turnover, barrier maintenance, and extracellular matrix synthesis [52]. Hypomethylation of repetitive elements increases genomic instability, while hypermethylation of promoter regions silences genes involved in antioxidant defense, DNA repair, and collagen production. Additionally, age-associated changes in histone acetylation and methylation shift chromatin toward a more repressive configuration, impairing the expression of genes essential for keratinocyte differentiation and fibroblast function [52]. These epigenetic alterations do not merely reflect aging, they actively drive it by reducing the skin’s capacity to respond to stress, repair damage, and maintain tissue architecture.

As biomolecular damage accumulates and gene-regulatory networks become increasingly disordered, the skin loses its ability to sustain homeostasis, leading to the characteristic phenotypes of aging: dermal thinning, collagen fragmentation, impaired barrier function, and heightened inflammatory susceptibility [39,40]. Collectively, these processes illustrate that molecular decay is not a passive byproduct of aging but an active driver of cutaneous degeneration. Thus, understanding these interconnected mechanisms provides a critical foundation for developing regenerative and longevity-focused aesthetic strategies that target the biology of aging rather than its superficial manifestations.

In this context, the multi-Omics technologies (genomics, epigenomics, transcriptomics, proteomics, metabolomics, and lipidomics) are essential for unraveling the molecular signatures that characterize skin across different age ranges [53,54,55,56]. By integrating these high-resolution datasets, multi-Omics approaches enable the identification of age-specific alterations in gene expression, chromatin state, protein turnover, mitochondrial metabolites, ECM components, and barrier lipids [53,54,55,56]. This system level profiling reveals not only the pathways that deteriorate with age but also the compensatory mechanisms that remain active, offering a precise map of the biological age of the skin rather than relying solely on chronological age. Complementing these laboratory-based insights, clinical skin assessment, encompassing biophysical measurements, imaging technologies, and morphological evaluation, provides a real-time appraisal of cutaneous function, including hydration, elasticity, pigmentation patterns, vascular changes, and barrier integrity [57,58]. When combined, multi-Omics profiling and clinical assessment create a comprehensive diagnostic framework that allows practitioners to determine the true physiological status of the skin and select the most appropriate, personalized intervention protocols. This integrative strategy enhances therapeutic precision, optimizes treatment sequencing, and ultimately improves the consistency and durability of aesthetic outcomes.

In addition, the accumulation of genotoxic load, arising from both replication-fork stress and UV-induced lesions, establishes a critical biological bottleneck that directly compromises cellular energy homeostasis.

Cellular energy homeostasis is fundamental to the maintenance of cutaneous structure and function, as the skin relies on high and continuous metabolic activity to support keratinocyte turnover, fibroblast-driven ECM synthesis, immune surveillance, and barrier repair [59,60,61]. At the core of this bioenergetic network are the mitochondria, which generate ATP through oxidative phosphorylation and orchestrate multiple signaling pathways essential for cellular survival, redox balance, and tissue regeneration [62,63,64]. However, mitochondria are uniquely vulnerable to molecular decay due to their proximity to the electron transport chain, the primary endogenous source of reactive oxygen species (ROS), and the limited repair capacity of mitochondrial DNA (mtDNA) [65,66]. As a result, mitochondrial dysfunction becomes both a driver and a consequence of the aging process, progressively undermining the skin’s ability to sustain homeostasis [59,60,61].

Intrinsic aging contributes to mitochondrial decline through the gradual accumulation of mtDNA mutations, replication errors, and oxidative lesions that impair electron transport chain efficiency [62,63,64]. Replication-fork stress, a hallmark of genomic instability, affects not only nuclear DNA but also mtDNA, leading to deletions, point mutations, and compromised transcription of mitochondrial genes [62,63,64].

These defects reduce ATP output and increase electron leakage, amplifying ROS production in a self-reinforcing cycle of oxidative damage [62,63,64,67,68]. Extrinsic factors, particularly UV radiation, further accelerate mitochondrial decay. UV exposure induces direct mtDNA photolesions, disrupts mitochondrial membrane potential, and generates high levels of ROS that oxidize lipids, proteins, and nucleic acids [69,70,71]. UV-driven oxidative stress also activates nuclear DNA damage responses, contributing to cellular senescence and impairing the cross-talk between the nucleus and mitochondria that is required for coordinated stress adaptation [69,70]. Together, intrinsic genomic instability and extrinsic photodamage converge to compromise mitochondrial integrity, reducing the bioenergetic capacity of keratinocytes and fibroblasts and impairing their regenerative potential [42,49,69].

The decline in mitochondrial function has profound consequences for cutaneous physiology. Reduced ATP availability limits fibroblast proliferation and collagen synthesis, slows keratinocyte turnover, and weakens barrier repair mechanisms [72,73]. Simultaneously, dysfunctional mitochondria release pro-inflammatory signals and promote the senescence-associated secretory phenotype (SASP), which amplifies ECM degradation, disrupts dermal–epidermal communication, and sustains chronic low-grade inflammation [74,75,76,77,78,79,80]. This bioenergetic collapse contributes directly to the clinical hallmarks of aging skin, including dermal thinning, loss of elasticity, impaired wound healing, uneven pigmentation, and increased sensitivity to environmental stressors [72,73].

From a clinical perspective, the bioenergetic status of the skin can be inferred through a combination of biophysical measurements, imaging technologies, and functional assessments [81,82]. Reduced mitochondrial performance manifests as diminished elasticity (cutometry), slower recovery after mechanical stress, increased transepidermal water loss (TEWL), impaired barrier function, and altered vascular responses [81,82]. High-resolution imaging may reveal textural irregularities, collagen fragmentation, and pigmentation patterns associated with oxidative stress [83,84]. When integrated with patient history, including UV exposure, lifestyle factors, and chronological versus biological age, these assessments allow practitioners to determine whether the skin is in a state of energetic deficit, oxidative overload, or senescence-driven dysfunction.

This diagnostic insight is essential for selecting the most appropriate aesthetic protocol. Skin exhibiting signs of mitochondrial decline or oxidative stress may benefit from regenerative and longevity-focused strategies that enhance cellular energy metabolism, support mitochondrial biogenesis, and reduce ROS burden before or alongside interventional procedures. Such approaches may include bioenergetic-supportive topicals, antioxidant-rich preconditioning, controlled photobiomodulation, or protocols designed to modulate senescence and improve ECM remodeling. By aligning treatment selection with the skin’s underlying bioenergetic and molecular status, clinicians can optimize procedural outcomes, enhance tissue resilience, and promote long-term functional rejuvenation rather than transient cosmetic correction.

## 3. Modulating the Senescent Phenotype to Enhance Regenerative Outcomes

Beyond the mitochondrial decay previously discussed, senescence is further driven by a wider array of genomic stressors, including telomere attrition and oncogene activation [85,86,87].

Senescence is initiated by diverse stressors such as: DNA damage, telomere attrition, oxidative stress, mitochondrial dysfunction, oncogene activation, and replication-fork collapse [85,86,87,88,89,90,91,92,93,94]. These insults activate canonical signaling pathways that converge on cell-cycle inhibitors, particularly: *CDKN2A* (p16^INK4a)—enforces G1 arrest by inhibiting CDK4/6 [90,91]; *CDKN1A* (p21^CIP1)—downstream of p53, halts the cell cycle in response to DNA damage [85,88,95]; *TP53* (p53)—orchestrates DNA damage responses and senescence induction [96,97,98]; and *RB1* (retinoblastoma protein)—maintains chromatin repression of proliferation genes [99,100,101].

Senescent cells also undergo profound metabolic and structural changes, including mitochondrial dysfunction and elevated ROS production [79,102]; epigenetic remodeling, such as heterochromatin formation (SAHF) [99,103]; altered proteostasis and lysosomal expansion (increased SA-β-gal activity) [86,104]; secretion of SASP factors, including IL-6, IL-8, MMP-1, MMP-3, GM-CSF, and TGF-β modulators [105,106]. The SASP is particularly detrimental in skin, as it promotes ECM breakdown, disrupts dermal–epidermal communication, recruits inflammatory cells, and propagates senescence to neighboring cells [105,106,107,108,109].

Although senescence is a molecular process, its consequences can be evaluated through clinical and biophysical assessments that reflect underlying cellular dysfunction. Key indicators include reduced elasticity and viscoelastic recovery (cutometry), TEWL indicating barrier impairment, delayed wound healing or slow recovery after controlled injury, textural roughness, fine lines, and dermal thinning via high-resolution imaging, pigmentary dysregulation associated with senescent melanocytes, vascular irregularities linked to chronic inflammation [110,111].

Multi-Omics technologies provide unprecedented resolution for characterizing the molecular landscape of senescent skin. These approaches enable the identification of age-specific and cell-type-specific senescence signatures, supporting the development of targeted interventions. Genomics reveals mutations, telomere shortening, and DNA damage patterns [112,113]. Epigenomics identifies methylation drift, chromatin remodeling, and senescence-associated epigenetic clocks [114]. Transcriptomics captures SASP gene expression, cell-cycle arrest markers, and mitochondrial stress responses [115,116]. Proteomics quantifies ECM-degrading enzymes, cytokines, and senescence-associated proteins [117,118]. Metabolomics and lipidomics uncover shifts in mitochondrial metabolites, ROS-related pathways, and barrier lipid composition [119,120]. Integrating these datasets allows researchers to define molecular phenotypes of senescence across different age groups, anatomical sites, and environmental exposures. This precision profiling supports the identification of novel therapeutic targets, including regulators of mitochondrial homeostasis, chromatin modifiers, and SASP mediators.

Emerging interventions aim not only to mask the visible signs of aging but to modulate or reverse the senescent phenotype. These strategies fall into three major categories:

1. Selective removal of senescent cells (senolytics): based on the use of senolytics agents that inhibit pro-survival pathways (SCAPs), leading to selective elimination of senescent cells through apoptosis [121,122,123,124].
2. Modulation of SASP without killing cells (senomorphics): based on the use of senomorphics agents that suppress inflammatory and degradative SASP components [125,126,127].
3. Mitochondrial and epigenetic rejuvenators: These strategies aim to restore cellular energy and reverse epigenetic drift [128,129,130,131].

To translate these agents (Table 1) into effective topical or procedural strategies, formulations often incorporate nanocarriers (liposomes, extracellular vesicles such as exosomes, and solid lipid nanoparticles) for enhanced penetration [132,133], hydrogel or polymeric delivery systems for sustained release [134,135,136]. In addition, these agents can be combined in personalized protocols integrating antioxidants, peptides, and controlled energy-based devices, or employed in pre-conditioning regimens to optimize mitochondrial and barrier function before aesthetic procedures [137,138]. Among these, polydeoxyribonucleotide (PDRN) has emerged as a valuable regenerative biopolymer for optimizing skin bed preparation due to its well-documented ability to enhance tissue repair, angiogenesis, and controlled inflammation [139,140,141]. As demonstrated in recent clinical and preclinical studies, PDRN activates the adenosine A2A receptor, leading to increased expression of VEGF, stimulation of fibroblast proliferation, and upregulation of collagen synthesis, mechanisms essential for preparing a biologically active and receptive wound bed [139,142]. In vitro data show that PDRN promotes keratinocyte and dermal fibroblast migration and proliferation [143], while in vivo models, including diabetic wound systems, confirm accelerated re-epithelialization, improved vascularization, and modulation of inflammatory cytokines, all of which contribute to a more functional and better-structured tissue substrate [144].

The integration of high-resolution multi-Omics offers a sophisticated roadmap for the development of precision “anti-aging cocktails.” By identifying the specific molecular signatures of senescence unique to a patient’s dermal niche, it is possible to design synergistic formulations that strategically combine senolytics for targeted clearance, senomorphics for inflammatory modulation, and NAD+ boosters to restore bioenergetic capacity. This multi-targeted approach ensures that the skin is supported across several tiers of molecular decay simultaneously, effectively neutralizing the feed-forward loops of aging. However, the transition from multi-Omic identification to clinical application necessitates a rigorous validation phase. It is crucial to evaluate the efficacy and safety of these complex formulations using advanced in vitro models that move beyond traditional monolayers. Implementing 3D cell culture systems and “skin-on-a-chip” technologies is essential, as these models more accurately replicate the spatial architecture, mechanical tension, and paracrine signaling of the human dermis. Only through such robust testing against multi-Omics-identified targets can we distinguish truly transformative regenerative therapies from speculative approaches, ensuring that personalized formulations deliver measurable structural restoration.

## 4. Biological Boundaries of Regeneration and the Role of Regenerative Aesthetics in Modulating the Cutaneous Microenvironment

The concept of regeneration in human tissues must be interpreted within the constraints of mammalian biology. True regeneration, defined as the complete restoration of tissue architecture, cellular identity, and functional capacity without fibrosis, is a phenomenon largely restricted to early developmental stages [188,189,190,191,192]. During fetal life, the human integumentary system exhibits a predominantly regenerative response, characterized by minimal inflammation, absence of fibrotic remodeling, and rapid re-establishment of normal ECM organization [193,194,195,196]. This capacity declines sharply after birth, when the wound-healing response becomes dominated by reparative mechanisms rather than regenerative ones [193,194,195,196].

In adult tissues, injury triggers a cascade involving acute inflammation, fibroblast activation, ECM deposition, and eventual scar formation [188,189,190,191,192]. While this reparative response is essential for survival, it inherently prioritizes rapid closure over functional restoration. The persistence of pro-inflammatory cytokines, oxidative stress, and senescent cell accumulation further shifts the tissue environment toward fibrosis and metabolic decline [188,189,190,191,192]. Thus, from a strict biological standpoint, adult humans do not regenerate tissue in the classical sense; they repair it through adaptive but imperfect processes [188,189,190,191,192].

Comparative biology provides valuable insights into the determinants of true regeneration. Organisms such as salamanders and newts retain lifelong regenerative abilities, enabling the restoration of limbs, spinal cord segments, and complex neuromuscular structures [192,197,198,199]. These species share several conserved features: tightly regulated inflammatory responses, transient and non-fibrotic ECM remodeling, maintenance of a permissive niche for progenitor cell activation, and the capacity to re-engage embryonic developmental programs [192,197,198,199]. Importantly, these processes are not driven by a single molecular trigger but by the orchestration of a favorable microenvironment that supports organized cellular proliferation and patterning [192,197,198,199].

This understanding has significant implications for the emerging field of regenerative aesthetics. Rather than attempting to induce true regeneration, which remains biologically unattainable in adult human skin, the goal is to approximate a more youthful, pro-regenerative microenvironment. This involves modulating key determinants of tissue quality, including chronic inflammation, ECM disorganization, cellular senescence, mitochondrial dysfunction, and impaired intercellular signaling [188,189,190,191,192]. By restoring a microenvironment that resembles the conditions of metabolically competent, younger tissue, aesthetic interventions can shift the balance of wound healing toward more functional and less fibrotic outcomes [193,194,195,196].

Current strategies in regenerative aesthetics, such as biostimulatory injectables, exosome-rich preparations, peptides, and bioactive scaffolds, aim to influence the cutaneous niche rather than merely inducing collagen deposition [2,10,200,201]. Their therapeutic value lies in promoting controlled remodeling, enhancing cellular communication, and reducing pathological inflammatory signaling [2,10,184,200,201,202,203,204]. Although these interventions do not achieve true regeneration, they can meaningfully improve tissue organization, resilience, and longevity by optimizing the biological context in which repair occurs [2,10,184,200,201,202,203,204].

In this sense, regenerative aesthetics represents a paradigm shift: from procedures that impose structural change to approaches that cultivate the conditions necessary for healthier, more adaptive tissue behavior. The focus moves from forcing regeneration to enabling the skin to repair itself in a manner that is more physiologically aligned with youthful tissue dynamics. This conceptual reframing is essential for advancing the field and for establishing realistic, biologically grounded expectations regarding the potential and limitations of cutaneous rejuvenation.

## 5. Regenerative Longevity Aesthetics Versus Conventional Aesthetic Interventions

While the pathophysiology of aging involves the complex interplay of intrinsic and extrinsic factors detailed in Section 2, conventional aesthetic interventions traditionally focus on the visible outcomes of these processes. These interventions can produce rapid, noticeable improvements and remain valuable tools for aesthetic enhancement. However, because they do not modify the cellular and molecular mechanisms that perpetuate aging, their benefits are inherently temporary [137,205,206,207]. Collagen degradation continues, oxidative stress accumulates, fibroblast activity declines, and the ECM becomes progressively disorganized. As a result, repeated treatments are required to maintain the desired aesthetic effect, reinforcing a cycle of correction rather than prevention. This approach, while effective in the short term, does not alter the biological trajectory of cutaneous aging [137,205,206,207].

In contrast, regenerative medicine-based strategies represent a paradigm shift toward biological durability and long-term tissue health. Regenerative longevity aesthetics aim to preserve or restore the skin’s intrinsic regenerative capacity by targeting the mechanisms that drive aging at their source. These interventions focus on enhancing cellular metabolism, improving mitochondrial efficiency, modulating inflammatory pathways, supporting stem cell function, and maintaining ECM integrity [208,209]. By doing so, they seek to slow or modulate the aging process itself rather than merely masking its manifestations.

Regenerative modalities, including bioactive peptides, growth-factor-rich formulations, exosomes, platelet-derived biomolecules, and energy-based devices applied in a regenerative rather than ablative manner, work by reinforcing these biological systems [2,208,209]. Their goal is not only to improve appearance but to sustain long-term skin vitality, resilience, and function. This approach aligns cosmetic practice with principles of longevity science, emphasizing outcomes that are both aesthetically favorable and biologically meaningful.

Crucially, regenerative interventions achieve their full potential only when integrated into a continuous, physiology-supportive skincare routine. Daily topical strategies, such as antioxidants, retinoids, barrier-supportive lipids, mitochondrial cofactors, and senescence-modulating actives (Table 1) help maintain dermal homeostasis between procedures [210,211]. These formulations counteract oxidative stress, support ECM remodeling, enhance epidermal turnover, and reinforce barrier integrity [210,211]. When combined with periodic regenerative treatments, they create a synergistic cycle in which skincare and procedures work together to sustain long-term skin health [210,211].

This integrated model acknowledges that skin aging is not a static event but an ongoing biological process requiring consistent support. Maintaining optimal physiology involves protecting the barrier, minimizing oxidative and inflammatory stress, stimulating balanced ECM remodeling, and preserving mitochondrial vitality [2,208,209]. Regenerative longevity aesthetics therefore shift the focus from temporary correction to long-term preservation and enhancement of skin function [2,208,209]. By addressing both the visible and biological dimensions of aging, this approach offers a more sustainable pathway to healthy, resilient, and aesthetically harmonious skin throughout the lifespan.

## 6. Skin Bed Preparation in Regenerative Aesthetics as a Core Pillar to Enhance the Skin’s Response to Therapeutic Interventions

Building on the previously defined framework, skin bed preparation is executed through targeted protocols that transition the skin from a catabolic to an anabolic state [210,212]. In doing so, this strategy creates a more favorable biological substrate for collagen induction, ECM remodeling, and tissue regeneration. For this reason, cutaneous bed preparation can be considered a fundamental phase of contemporary regenerative aesthetic protocols, contributing to enhanced efficacy, safety, and durability of aesthetic procedures [210,212,213,214,215,216,217].

The rationale for this approach is based on the understanding that aged skin presents significant structural and metabolic alterations, including reduced mitochondrial efficiency, impaired barrier function, chronic low-grade inflammation, decreased fibroblast activity, and fragmentation of the ECM [210,212,213,214,215,216,217]. These changes limit the ability of cutaneous tissue to respond adequately to treatments that depend on fibroblast activation, collagen synthesis, and the integrity of the dermal microenvironment. Thus, by restoring metabolic balance, reinforcing barrier integrity, and modulating inflammatory pathways prior to intervention, skin bed preparation significantly increases clinical efficacy, the predictability of outcomes, and the safety of regenerative procedures [210,212].

Strategies for preparing the skin bed generally involve the use of targeted topical and bioactive formulations, including antioxidants, retinoids, mitochondrial cofactors, signaling peptides, agents that modulate cellular senescence, barrier-restoring lipids, as well as regenerative biomolecules such as polydeoxyribonucleotides (PDRN) and exosome-based preparations [210,212]. These agents act synergistically to enhance cellular resilience, reduce oxidative stress, support inflammatory homeostasis, and stimulate tissue regeneration processes. In addition, they help normalize keratinocyte turnover, promote the reorganization of the extracellular matrix, and optimize hydration and lipid organization within the epidermis [210,212]. When applied consistently, they create a microenvironment conducive to regeneration, allowing procedures such as energy-based devices, biostimulatory injectables, platelet-derived biomolecules, or exosome-rich preparations to achieve deeper and more sustained biological effects [210,212].

In addition to topical approaches, skin bed preparation may include non-ablative energy-based modalities used in a preparatory manner to stimulate mild fibroblast activation, enhance microcirculation, and promote controlled remodeling without inducing significant inflammation [218,219,220,221]. This pre-conditioning effect increases the skin’s receptivity to subsequent regenerative treatments, aligning procedural timing with the skin’s biological rhythms and repair capacity [218,219,220].

Furthermore, skin bed preparation strategies can also be applied together with conventional aesthetic treatments. As an example, our recent clinical trial demonstrated that combining calcium hydroxyapatite (CaHa) with a regenerative solution enriched in targeted cosmetic actives can significantly potentiate the outcomes typically achieved with CaHA alone [222]. By incorporating amino acids, minerals, antioxidants, and metabolic cofactors into the diluent, the formulation created a more supportive biochemical environment for fibroblast activation, resulting in enhanced neocollagenesis and more organized dermal remodeling over a 90-day period [222]. Compared with standard saline dilution, this regenerative solution suggests providing the necessary building blocks and signal that an aged, nutrient-depleted microenvironment can no longer provide on its own [222]. These findings reinforce the growing concept that preparing the skin with bioactive components, and integrating them into procedural workflows, can amplify the efficacy of established injectables, ultimately elevating clinical results and promoting more comprehensive tissue rejuvenation.

Thus, skin bed preparation also reinforces the concept that regenerative longevity aesthetics is not a single procedure but a continuous, physiology-centered practice. Maintaining the benefits of regenerative treatments requires ongoing support of the skin’s intrinsic functions. Daily skincare becomes a biological maintenance system, sustaining barrier function, mitigating oxidative and inflammatory stress, and preserving ECM integrity, while periodic regenerative procedures provide targeted boosts to cellular and structural renewal [210,212]. This cyclical model mirrors principles of preventive and longevity medicine, emphasizing consistency, biological alignment, and long-term tissue stewardship.

Ultimately, skin bed preparation shifts the aesthetic paradigm from reactive correction to proactive regeneration. By preparing the skin to respond optimally to interventions and maintaining its physiological balance between treatments, skin bed preparation enhances both the immediate and long-term outcomes of regenerative longevity aesthetics. It ensures that aesthetic improvements are not only visible but also rooted in durable biological change, supporting healthier, more resilient, and more youthful skin over time.

## 7. NAD^+^ Boosters (NMN, NR, NAD^+^ Precursors) and Senotherapeutic Peptides as a Fundamental Strategy for Skin Bed Preparation

As skin ages, there is a progressive decline in mitochondrial efficiency, decreasing by 5% to 8% in energy production per decade, which leads to an accumulation of ROS and the permanent arrest of cell division known as cellular senescence [128,223]. This energy depletion triggers the SASP, a state where fibroblasts downregulate pro-collagen genes (COL1A1, COL3A3) and overexpress MMPs, effectively degrading the ECM from within [224,225]. Without sufficient adenosine triphosphate (ATP), dermal fibroblasts lose the cytoskeletal contractile force required to resist mechanical stress and respond adaptively to aesthetic interventions [226,227]. To mitigate this, advanced dermatological formulations now prioritize active molecules such as nicotinamide adenine dinucleotide (NAD+) precursors [170,228], urolithin A [229,230], and senotherapeutic peptides (e.g., OS-01), which specifically target the hallmarks of aging to restore cellular energy [231,232].

The decline in intracellular NAD^+^ levels is increasingly recognized as a hallmark of cutaneous aging, contributing to impaired mitochondrial respiration, reduced genomic maintenance, and diminished cellular resilience [172,233,234]. In the context of regenerative longevity aesthetics, strategies that restore or enhance NAD^+^ availability, through topical or transdermal delivery of precursors such as nicotinamide mononucleotide (NMN), nicotinamide riboside (NR), or stabilized NAD^+^ derivatives, are emerging as promising tools for metabolic skin bed preparation prior to aesthetic interventions [172,233,235].

From a cosmetic science perspective, NAD^+^ boosters function as metabolic enhancers that support the skin’s ability to undergo controlled regeneration. Their relevance lies not in therapeutic claims but in their capacity to optimize the biological environment in which aesthetic procedures operate. By elevating NAD^+^ pools, these molecules influence several pathways central to cutaneous structure, function, and appearance, through:

1. Enhancement of cellular bioenergetic: keratinocytes and fibroblasts rely heavily on mitochondrial ATP production to sustain proliferation, differentiation, and ECM synthesis [61,78,128]. NAD^+^ precursors can support oxidative phosphorylation efficiency, thereby improving the energetic capacity of these cells during the remodeling phase that follows procedures such as microneedling, fractional lasers, or biostimulatory injectables [172,233,234]. Enhanced ATP availability may facilitate more coordinated collagen deposition, improved barrier recovery, and more efficient turnover of damaged cellular components [172,233,234].
2. Support of genomic maintenance and cellular quality control: Sirtuins (NAD^+^-dependent deacetylases) play a central role in chromatin regulation, DNA repair coordination, and cellular stress adaptation [236,237,238,239]. Age-related NAD^+^ depletion reduces sirtuin activity, contributing to genomic instability and accumulation of dysfunctional cells [238,240,241]. By increasing NAD^+^ availability, NMN and NR may help maintain a more stable genomic environment, supporting the skin’s capacity to respond predictably to controlled micro-injury induced by aesthetic procedures [242,243]. This genomic support aligns with the goals of regenerative longevity aesthetics, which emphasize long-term tissue quality rather than transient cosmetic correction.
3. Reduction in oxidative and inflammatory burden: Oxidative stress and low-grade inflammation are major contributors to ECM degradation and impaired wound-healing dynamics [244,245]. NAD^+^ boosters can influence redox homeostasis by supporting mitochondrial antioxidant systems and reducing ROS generation [246,247].
4. Promotion of mitochondrial biogenesis and cellular resilience: Mitochondrial dysfunction is a defining feature of aged skin, contributing to reduced fibroblast contractility, impaired ECM synthesis, and slower epidermal turnover [62,130]. NAD^+^ precursors may activate pathways associated with mitochondrial biogenesis and mitophagy, helping maintain a healthier mitochondrial network [172,248,249]. These effects increase the skin’s resilience, ensuring that fibroblasts are in a high-metabolic state to maximize neocollagenesis following energy-based or injectable treatments [250,251]. By addressing the phenotypic variability of the skin’s response early, practitioners can significantly mitigate the risks of delayed wound healing [252,253].

Thus, the incorporation of NAD^+^ boosters into aesthetic protocols represents a significant evolution in strategies aimed at optimizing the cutaneous microenvironment before, during, and after regenerative procedures. Rather than functioning as isolated bioactive agents, NAD^+^ precursors, such as nicotinamide riboside (NR), nicotinamide mononucleotide (NMN), and niacinamide, exert their greatest impact when integrated into a broader biochemical framework that includes peptides, antioxidants, barrier-supportive lipids, and other skin-conditioning compounds [254,255]. This multimodal approach aligns with contemporary evidence indicating that tissue regeneration is not driven by a single molecular pathway but by the coordinated optimization of cellular metabolism, redox balance, and ECM homeostasis [207,256,257,258].

NAD^+^ boosters enhance intracellular energy availability, support mitochondrial function [172,248,249], and modulate sirtuin activity, mechanisms that collectively improve cellular resilience and reduce the burden of oxidative and metabolic stress [242,243,259]. However, these benefits are amplified when the surrounding microenvironment is simultaneously conditioned to support efficient repair. Peptides and/or EVs with signaling or matrikine activity can enhance fibroblast communication and ECM remodeling; antioxidants mitigate reactive oxygen species that otherwise deplete NAD^+^ pools; and barrier-supportive lipids stabilize the stratum corneum, reducing transepidermal water loss and inflammatory signaling [179,207]. When used together, these agents create a biologically favorable milieu that supports more organized and functional tissue remodeling.

## 8. The Peptide-Primed Matrix: Optimizing the “Skin Bed Preparation” for Aesthetic Success

The shift in aesthetic medicine from reactive “filling” to proactive “regeneration” has repositioned topically applied and injectable peptides as critical components of “skin bed preparation”. In this context, peptides are no longer viewed merely as anti-aging adjuncts but as indispensable biochemical primers that optimize the dermal microenvironment prior to and following minimally invasive procedures [260,261,262,263].

At the molecular level, the “skin bed preparation” represents a complex interplay between fibroblasts, the ECM, and the microvascular network. Peptides act as biomimetic ligands that modulate these components through specific cellular receptors.

In vitro studies into signal peptides, particularly the matrikine-mimetic palmitoyl pentapeptide-4, has demonstrated via in vitro human dermal fibroblast (HDF) cultures a dose-dependent increase in the mRNA expression of type I collagen, type III collagen, and fibronectin [168]. Furthermore, the copper-binding peptide GHK-Cu (glycyl-L-histidyl-L-lysine) has shown significant capacity to modulate matrix metalloproteinases (MMPs) and their inhibitors (TIMPs). By balancing the proteolytic environment, GHK-Cu prepares the skin bed by removing fragmented, non-functional collagen, thereby clearing the “scaffold” for new neo-collagenesis induced by aesthetic interventions [264,265].

Clinical trials utilizing split-face methodologies have highlighted the impact of peptide-based SBP. Patients pre-treated with a combination of palmitoyl tetrapeptide-7 and palmitoyl tripeptide-1 for 28 days prior to ablative procedures exhibited enhanced barrier recovery and inflammatory modulation [266].

The role of peptides extends into the maintenance phase of the skin bed. By inhibiting cellular senescence markers, such as progerin, peptides like trifluoroacetyl tripeptide-2 help maintain the structural integrity achieved through clinical procedures [267]. This biological preservation ensures that the newly synthesized collagen remains organized and functional, delaying the return of rhytids and skin laxity.

## 9. The Dual Role of Extracellular Vesicles in “Skin Bed Preparation” and Regenerative Aesthetic: Multi-Molecular Delivery for Enhanced Aesthetic Outcomes

The paradigm of regenerative aesthetics is shifting from simple topical supplementation to “cell-free” signaling modulation, driven primarily by the transformative potential of extracellular vesicles (EVs) [2,268,269]. These nano-sized vesicles (30–1000 nm) function as sophisticated delivery vehicles that simultaneously transfer a complex “intercellular language” comprising proteins, lipids, and nucleic acids (mRNA, miRNA) [2,184,202,270,271]. Unlike single-molecule therapeutics, the heterogeneous cargo of EVs enables the orchestration of synchronized biological responses in recipient cells, a process increasingly recognized as skin bed preparation. By modulating the local microenvironment through specialized cell-to-cell communication, EVs prime the dermal and epidermal layers to be more responsive to subsequent aesthetic interventions, such as microneedling, laser resurfacing, or injectable fillers [2,268,269].

The clinical utility of this bio-priming depends heavily on the EV source, as the transcriptomic and proteomic signatures vary significantly between human, plant, and bacterial origins [2].

Human-derived EVs, particularly those isolated from Mesenchymal Stem Cells (MSCs) or Platelet-Rich Plasma (PRP), represent the current gold standard in regenerative potential [2]. Their intrinsic cargo includes essential growth factors (VEGF, TGF-β, EGF) and anti-inflammatory cytokines that directly stimulate fibroblast proliferation and neocollagenesis [187,272]. However, MSC-derived EVs integration into the global market is severely hindered by regulatory prohibitions in several jurisdictions (e.g., European and US cosmetic regulations) due to concerns over viral transmission and ethical sourcing [2]. This regulatory bottleneck has catalyzed the search for alternative sources that can mimic these regenerative signals without the associated biosecurity risks [2].

In this context, plant-derived EVs (PDEVs) have emerged as a highly biocompatible and sustainable alternative [2,273]. These vesicles are rich in secondary metabolites and antioxidants that offer robust protection against photoaging and oxidative stress [2,273,274,275,276]. While PDEVs are favored for their safety and ease of large-scale production, their evolutionary distance from human physiology may limit their ability to induce specific human-like transcriptional changes compared to mammalian EVs [2]. Similarly, bacterium-derived EVs (BEVs), specifically those from probiotic strains offer unique immunomodulatory benefits [2]. These vesicles can enhance the skin barrier and modulate the local microbiome; however, the potential for pro-inflammatory reactions triggered by bacterial surface antigens (like LPS) necessitates rigorous purification protocols to ensure clinical safety [2,269,277,278].

The strategic application of these various EV populations facilitates a state of tissue readiness. By transferring regulatory miRNAs, mRNA and signaling ligands, EVs reconfigure the recipient cell’s metabolic and secretory profile, reducing chronic inflammation and optimizing the ECM [2,279,280]. This preparatory phase, the priming of the skin bed, ensures that when a secondary aesthetic treatment is applied, the tissue is not merely a passive recipient but an active, responsive environment. Consequently, the simultaneous transfer of multiple molecular signals via EVs does not just repair the skin; it fundamentally re-engineers cellular communication to maximize the longevity and efficacy of regenerative aesthetic outcomes.

Despite the significant potential of human-, plant-, and bacteria-derived EVs in skin bed preparation, several unresolved technical challenges limit their immediate translational maturity. The inherent cargo heterogeneity and batch-to-batch variability, driven by the physiological state of the donor cells and the isolation method used (e.g., ultracentrifugation vs. tangential flow filtration), pose substantial hurdles for therapeutic standardization [2].

Furthermore, the clinical translation of EVs into the aesthetic market is fundamentally constrained by their inherent structural fragility and the challenges of maintaining cargo stability within topical formulations [2]. As lipid-bilayered nanostructures, EVs are susceptible to premature degradation, fusion, or cargo leakage when exposed to the varying pH levels, surfactants, and temperature fluctuations typical of cosmetic manufacturing and shelf storage [2]. Traditional aqueous environments often fail to preserve the integrity of the delicate miRNA and protein signatures that drive the skin bed preparation effect. To overcome these limitations, advanced stabilization strategies, such as lyophilization (freeze-drying) to maintain a metabolic ‘standby’ state, or encapsulation within secondary delivery systems like hydrogels or solid lipid nanoparticles, are increasingly being explored [2]. These techniques aim to ensure that the EVs remain structurally intact and biologically active until the point of application, thereby providing the consistent and predictable molecular signaling required for effective skin bed preparation.

## 10. Conclusions

The evolution of the global aesthetic market toward regenerative longevity aesthetics marks a definitive shift from temporary, volume-based corrections to the biological preservation of tissue function. As this review has demonstrated, the success of modern aesthetic interventions is no longer solely dependent on the technical execution of a procedure, but on the physiological status of the cutaneous environment.

The integration of skin bed preparation into clinical protocols addresses the fundamental hallmarks of aging, molecular decay, mitochondrial dysfunction, and cellular senescence, that otherwise limit the skin’s regenerative capacity.

While NAD^+^ precursors, peptides, PDRN, and exosome-based preparations are central to the regenerative paradigm, it is critical to distinguish their operational and regulatory identities to avoid the perception of a unified therapeutic category. For instance, peptides and NAD^+^ precursors (such as NMN) are primarily utilized as topical cosmeceuticals or nutritional supplements with a focus on metabolic support and enzymatic co-factors. Conversely, PDRN (Polydeoxyribonucleotide) often occupies a medical device or pharmaceutical category depending on the jurisdiction, generally requiring intradermal delivery to trigger adenosine A2A receptor-mediated tissue repair. Exosome-based preparations represent a more complex biological frontier; they are currently categorized under advanced therapy medicinal products (ATMPs) or ‘cell-free’ biologics, facing the most stringent regulatory scrutiny regarding isolation purity and standardization. Furthermore, while the evidence base for peptides and PDRN is supported by established clinical trials, the application of exosomes in aesthetics remains largely driven by emerging mechanistic studies and pilot clinical data. Recognizing these distinctions is essential for a precise clinical application of skin bed preparation.

Furthermore, the synergy between advanced multi-Omics profiling and targeted topical maintenance establishes a new standard for personalized aesthetics. This comprehensive diagnostic and therapeutic framework ensure that aesthetic outcomes are not only visually harmonious but also biologically durable. Ultimately, prioritizing the health of the “skin bed” allows for a transition from reactive correction to proactive regeneration, optimizing patient safety, healing velocity, and long-term psychosocial well-being in an increasingly image-conscious society.

## Figures and Tables

**Table 1:** Key anti-senescence agents and targeted biological pathways in skin aging.

Class of Agents	Agents	Mechanism of Action	Reference
**Senolytics**	quercetin	modulates PI3K/AKT and reduces SASP factors	[123,145]
	fisetin	induces apoptosis in senescent fibroblasts	[146,147,148,149]
	Dasatinib and quercetin (D + Q)	synergistically clears senescent cells	[145,150]
	Navitoclax (BCL-2 inhibitor)	targets anti-apoptotic pathways upregulated in senescence	[151,152,153]
**Senomorphics**	retinoids	regulate gene expression, enhance turnover, reduce MMPs	[154,155,156,157]
	niacinamide	improves mitochondrial function and reduces oxidative stress	[158]
	resveratrol and polyphenols	activate SIRT1, modulate NF-κB, and reduce SASP	[159,160,161,162]
	peptides (e.g., GHK-Cu	stimulate ECM repair and reduce inflammatory signaling	[163,164]
	Lactosomes	Act as a cell signaling messenger, promoting delivery of bioactive molecules, supporting epidermal renewal and anti-inflammatory responses, improving skin barrier strengthening	[165,166]
	Palmitoyl Pentapeptide-4	Mimics the natural breakdown products of collagen, trigging a feedback loop that stimulates the cell to initiate the synthesis of new structural components such as collagen type I and IV, fibronectin, and hyaluronic acid	[167,168,169]
**Mitochondrial and epigenetic rejuvenators**	NAD^+^ boosters (NMN, NR)	enhance mitochondrial function and DNA repair	[170,171,172]
	CoQ10 and mitochondrial antioxidants	reduce ROS and improve ATP production	[129,173]
	Peptides (e.g., MOTS-c, SS-31)	mitochondrial biogenesis	[174,175,176,177,178,179,180,181]
	Epigenetic modulators	HDAC inhibitors or sirtuin activators that restore youthful gene expression patterns	[52,182,183]
**Extracellular vesicles**	Exosomes	Bioactive nanocarriers that deliver proteins, lipids, nucleic acids, and metabolites to skin cells, triggering regenerative, anti-inflammatory, and pigmentation-modulating effects, stimulating collagen and ECM production and accelerating wound healing	[184,185,186,187]

## Data Availability

No new data were created or analyzed in this study.
